# Disorder‐specific attachment characteristics and experiences of childhood abuse and neglect in adolescents with anorexia nervosa and a major depressive episode

**DOI:** 10.1002/cpp.2324

**Published:** 2018-09-14

**Authors:** Manuela Gander, Kathrin Sevecke, Anna Buchheim

**Affiliations:** ^1^ Department of Child and Adolescent Psychiatry Medical University of Innsbruck Innsbruck Austria; ^2^ Institute of Psychology University of Innsbruck Innsbruck Austria

**Keywords:** adolescence, anorexia nervosa, attachment, childhood trauma, major depressive episode

## Abstract

For the first time, the present study investigates disorder‐specific attachment characteristics and childhood trauma in adolescent inpatients with anorexia nervosa (*n* = 30, girls/boys: 28/2, age: *M* = 14.84, *SD* = 1.20), a major depressive episode (*n* = 30, girls/boys: 27/3, age: *M* = 15.14, *SD* = 1.50), and controls (*n* = 60, girls/boys: 44/16, age: *M* = 16.10, *SD* = 1.20). We used the Structured Clinical Interview to diagnose Axis I disorders, the Adult Attachment Projective Picture System to classify attachment representations, and the Childhood Trauma Questionnaire to assess child maltreatment. Our findings demonstrate an overrepresentation of the unresolved attachment status in the patient samples. A one‐way analysis of variance succeeded by Bonferroni post hoc tests indicated that adolescents with anorexia nervosa show more isolation and dissolution of boundaries between life and death when confronted with situations of solitude. Although they report moderate to severe levels of traumatic childhood experiences, they tend to minimize those. Adolescents with a major depressive episode report higher levels of emotional abuse and neglect in their childhood, leaving them in a state of failed protection and danger during attachment distress. Integrating these attachment‐related characteristics into specific psychotherapeutic interventions might be associated with a better outcome in that age group.

**Key Practitioner Message**

- The findings from the present study demonstrate an overrepresentation of the unresolved attachment status in adolescents with anorexia nervosa and a major depressive episode
- Adolescents with anorexia nervosa showed more unresolved themes that indicate isolation and dissolution of boundaries between life and death when confronted with attachment‐related stressors
- Adolescents with a major depressive episode reported the highest levels of emotional neglect and abuse in childhood that is also reflected in a fair amount of isolation, danger, and failed protection in their attachment narratives
- Taking a closer look at the unresolved attachment status and traumatic material might contribute to a better understanding of how attachment‐related aspects mediate early trauma and adolescent psychopathology
- Clinicians who treat adolescents with a history of trauma might benefit from assessing attachment patterns and analyse attachment‐related characteristics as they provide a far‐reaching insight into the psychological functioning within and beyond the family system

## 1 INTRODUCTION

### 1.1 Childhood trauma and neglect

Child abuse and neglect can result in a wide range of adverse consequences for adolescents and young adults. Among those are early manifestations of mental health problems. In particular, anorexia nervosa (AN) and depression in adolescent age groups seem to be significantly associated to child maltreatment. Research in the field of traumatic experiences distinguishes between the effects of non‐interpersonal traumas (i.e., accidents, physical illness, and natural disasters), interpersonal traumas (i.e., child abuse and maltreatment, domestic violence, and sexual abuse), and adverse childhood circumstances (i.e., difficult upbringing conditions). Recently published studies demonstrated that patients with an eating disorder (ED) often report emotional abuse, mental health problems in parents, or parental divorce during their upbringing (Lejonclou, Nilsson, & Holmqvist, 2014; Maxwell et al., 2017). It is also suggested that sexual trauma is related more often to bulimia nervosa (BN) or AN binge‐eating/purging type, than to AN, restrictive type (AN‐R; Brewerton, 2007; Jaite et al., 2012). Even though theorists propose that sexual and physical traumatization represent non‐specific risk factors (Lejonclou et al., 2014), clinicians often face histories of various abusive experiences in their patients with ED. Similarly, longitudinal studies found that adolescents experiencing abuse and neglect in childhood were three times more likely to develop a depressive disorder than those who had not been maltreated (Harkness, Lumley, & Truss, 2008). However, research findings on childhood trauma often use different instruments to assess traumatic experiences, and thus, they often define traumatic experiences in a different way. For example, the definitions of sexual abuse can vary from broad ones considering one‐time incidents of being exposed to a perpetrator to narrow ones that only include experiences of subject genital contact. Other studies do not use severity ratings of traumatic experiences but speak of abused or non‐abused dichotomies. These methodological and definitional differences might be one reason for some inconsistent findings on links between childhood trauma and psychopathological symptoms in adolescents and adults (Jaite et al., 2012; Kong & Kunsook, 2009; Lejonclou et al., 2014). The present study used the definitions provided by Bernstein and Fink (1998) based on the childhood trauma literature: Emotional abuse includes all forms of verbal assaults and humiliating or threatening behaviour towards a child by an older person; physical abuse is defined as an intentional behaviour by an adult that puts a child at risk or causes an injury or other physical suffering; sexual abuse refers to sexual behaviour towards a child (including sexual conduct, contact, and coercion); emotional neglect refers to the caregivers' failure to provide comfort, love, encouragement, and support; and physical neglect refers to the caregivers' failure to provide physical needs including food, safety, shelter, and health.

To date, hardly any research paid attention to possible mechanisms relating experiences of child abuse and neglect to AN and depression (Tasca et al., 2013). In this respect, the quality of an attachment relationship to a primary caregiver might play a key role in this association as it affects the way individuals deal with the exposure to childhood trauma (Solomon & George, 2011). A secure quality of attachment usually leads to greater resilience and helps adolescents when confronted with traumatic experiences. On the contrary, an insecure or even an unresolved attachment quality might lead to feelings of helplessness and being caught in these experiences as attachment figures do not help to restore safety or integration of these experiences (Laible, 2007). Therefore, an insecure or unresolved attachment status in adolescents might provide a fertile ground for the development of psychopathology in response to experiences of neglect and abuse in early childhood.

### 1.2 Attachment characteristics

In the field of AN, numerous studies focusing on attachment issues and family dynamics in ED have been published in the last decades (Gander, Sevecke, & Buchheim, 2015). Among them are some reviews summarizing research findings primarily in adults (O'Shaughnessy & Dallos, 2009; Tasca & Balfour, 2014; Tasca, Ritchie, & Balfour, 2011; Zachrisson & Skårderud, 2010) and also in adolescents (Gander et al., 2015). The majority of studies regarding adolescents are using self‐report measures that assess a person's conscious appraisals of behaviours and feelings in attachment relationships. In sum, these studies found that insecure attachment styles are overrepresented in adolescent girls with AN. Furthermore, they suggest that symptom severity and a worse treatment outcome are significantly associated with an anxious attachment style in adolescent and young adult patients with AN that is characterized by a high sensitivity to relationship loss and rejection, a lot of anger and preoccupation in attachment relationships, and a hyper‐activation of their attachment behaviour (De Paoli, Fuller‐Tyszkiewicz, & Krug, 2017; O'Shaughnessy & Dallos, 2009).

Concerning the relationship between childhood trauma and adult ED psychopathology, research implying attachment constructs has received attention in recent years. Studies found that insecure‐avoidant and insecure‐anxious attachment styles can partly explain this association in undergraduate students with anorexic and/or bulimic symptoms (Burns, Fischer, Jackson, & Harding, 2012) and adults with eating pathology (Tasca et al., 2013). However, more research is needed before strong conclusions can be drawn regarding the impact of early trauma on later AN. Furthermore, no study to date explored these issues in an adolescent age group. In this respect, studies incorporating attachment interviews that allow the assessment of unconscious defensive strategies (Ravitz, Maunder, Hunter, Sthankiya, & Lancee, 2010) and the classification of the unresolved attachment status (U)—a category that is associated with severe neglect and abuse by caregivers (Buchheim, Gander, & Juen, 2014; George & West, 2012)—might make important contributions to this field.

Narrative interviews like the Adult Attachment Interview (Hesse, 2008) or the Adult Attachment Projective Picture System (AAP; George & West, 2012) emphasize mental representations of attachment. In these interviews, people talk about attachment situations, and the patterns of responses are analysed in terms of unconscious defensive processes. The low convergent validity between self‐report and interview measurements suggests that they both measure different facets of attachment, especially in regard to loss, abuse, and neglect (Ravitz et al., 2010).

Based on the overrepresentation of the unresolved pattern especially in clinical populations (Bakermans‐Kranenburg & van IJzendoorn, 2009), researchers have increasingly called for a more systematic investigation of this attachment pattern and the nature of these traumatizing events related to attachment. First studies that systematically delved deeper into the unresolved status in adult borderline personality disorders and anxiety disorders reveal interesting disorder‐specific differences on the nature of attachment trauma (Buchheim et al., 2008, 2012, 2018; Buchheim & George, 2001).

The most striking result indicates a predominance of the unresolved pattern in adults with AN (Delvecchio, Di Riso, Salcuni, Lis, & George, 2014; Ringer & Crittenden, 2007; Von Wietersheim, Holzinger, Zhou, & Pokorny, 2014; Ward et al., 2001). The very few studies in this field found a lot of traumatic materials including eerie, evil, or surreal elements (Delvecchio et al., 2014), a lack of resolution of trauma and loss related to the mothers and also hidden family conflict between the parents (Dallos & Denford, 2008; 2007). To date, two studies have analysed the attachment narratives of daughters with AN and their mothers (Ringer & Crittenden, 2007; Ward et al., 2001). Despite the rather small sample size, they demonstrated an even higher rate of the unresolved pattern in the mothers than their adult daughters with ED in respect to loss of attachment figures and severe dangers in their past. The most outstanding feature of the mothers' discourse was that they did not want their daughters to be affected by their trauma (Ringer & Crittenden, 2007). Based on these preliminary results, it could be assumed that even though the mothers want to have a special closeness to their daughters in order to protect them from their past traumatic experiences, they retreat and become unavailable in situations their daughters need them the most (Gander et al., 2015). Thereby, they might superimpose their own needs on those of their daughters, resulting in a failed autonomy as their daughters never learned to differentiate their own needs. This might also explain why attachment studies in adults found problematic mother–daughter relationships characterized by a loving but at the same time rejecting, neglecting, and role‐reversing stance (Barone & Guiducci, 2009; Ringer & Crittenden, 2007). Furthermore, there is evidence that indicates an association between a negative perception of the family and emotional instability in adults with ED (Münch, Hunger, & Schweitzer, 2016).

However, hardly anything is known about the nature of these traumatizing experiences in daughters. Therefore, the first step is to examine disorder‐specific attachment characteristics and traumatic experiences in more detail. The present study expands the developments from Buchheim and George (Buchheim et al., 2008; Buchheim & George, 2001), who analysed specific markers of traumatic dysregulation in different mental disorders, by investigating these issues for the first time in an adolescent age group with AN. Furthermore, the majority of existing research findings is based on mixed ED populations including BN or binge eating disorder (BED), but they often do not differentiate between ED subgroups (Münch et al., 2016). However, some studies suggest that patients with AN‐R probably differ from patients with BN or BED in terms of their traumatic childhood experiences or family dynamics (Brewerton, 2007; Jaite et al., 2012). These differences might also be relevant for attachment‐related characteristics. Given that we want to identify disorder‐specific attachment characteristics, we therefore focused our research exclusively on adolescents with AN‐R. This new methodology combined with a very concentrated focus on AN might help to elucidate a deeper understanding of the role of attachment and traumatic childhood experiences of this disorder in adolescence.

In addition, we will compare adolescents with AN to an adolescent control group and another clinical group of adolescents with a major depressive episode (MDE) who do not show any signs of ED symptomatology. We chose to compare adolescent patients with AN and patients with an MDE for several reasons. First, an MDE has a prevalence rate of approximately 8% and is one of the most common psychiatric disorders in adolescence. Second, adult attachment studies in depression demonstrate that they rather show an overrepresentation of insecure attachment representations instead of the unresolved pattern (Bakermans‐Kranenburg & van IJzendoorn, 2009; Malik, Wells, & Wittkowski, 2014). Some studies report that the insecure‐preoccupied attachment group is very prevalent in depression (West & George, 2002), whereas others found the insecure‐dismissing attachment pattern primarily in episodically depressed and dysthymic patients (Dozier, Stovall‐McClough, & Albus, 2008). Due to the lower prevalence rate of unresolved attachment patterns in patients with depression, authors suggest that the origins of depressive disorders might be suspected in other attachment‐related roots. For example, Bakermans‐Kranenburg and van IJzendoorn (2009) state that child rearing or interpersonal issues might be more relevant for these patients than abuse or attachment trauma. Surprisingly however, recent research studies found that more than half of the participants with chronic depression had an unresolved status (Buchheim et al., 2018; Fischer‐Kern, Nolte, Kadi, & Naderer, 2014; Fonagy et al., 1996; Juen, Arnold, Meissner, Nolte, & Buchheim, 2013). These findings are in line with studies in children who found that disorganized attachment representations are significantly associated with clinical‐range depressive symptoms (Goodman, Stroh, & Valdez, 2012; Gullone, Ollendick, & King, 2006). Although a considerable amount of studies based on self‐report attachment measures have established a link between insecure attachment styles and depressive symptoms in adolescent age groups (Brenning, Soenens, Braet, & Bosmans, 2012; Puissant, Gauthier, & Van Oirbeek, 2011), no study to date has used a narrative measurement to examine the unresolved attachment status in relation to experiences of childhood trauma in adolescents with a depressive disorder.

### 1.3 The aims of the study

The present study aims to investigate the differences in disorder‐specific attachment characteristics and experiences of childhood abuse and neglect in adolescents with AN and an MDE compared with controls. In concordance with the findings from the reviewed research papers in adult samples, we postulate the following hypotheses for adolescents:

1. We assume an overrepresentation of the unresolved category in the patient samples compared with the adolescent control group with no history of psychiatric disorders.
2. Concerning attachment distributions, we believe that the group of patients with AN will show a higher prevalence rate of unresolved patterns compared with the group of patients with an MDE.
3. We suppose that there will be differences in attachment‐related defences and unresolved themes between the two clinical groups and the control group.
4. We expect that both patients with an MDE and patients with AN demonstrate higher scores on subjectively traumatic experiences than the control group. In this respect, we think that adolescents with depression score higher on emotional abuse and neglect than adolescents with AN, who rather tend to minimize their traumatic experiences.

## 2 METHOD AND DESIGN

### 2.1 Participants

Adolescents diagnosed with an ED or a major depressive disorder who attended the inpatient unit at the Department of Child and Adolescent Psychiatry at the Medical University of Innsbruck were invited to participate. A total of 126 consecutive inpatient adolescents were asked to participate. In line with previous studies using narrative attachment measurements in clinical patients who had groups of 60 (Ringer & Crittenden, 2007) or even less (i.e., 51 in the Delvecchio et al., 2014, study or 20 patients in the Ward et al., 2001, study), we chose to include 60 clinical patients and an equal number of nonclinical adolescents for the control group. As this study focuses on adolescent age groups and some of our assessment measurements are designed for adolescents not younger than 13 years of age, we included participants from 13.0 to 17.9 years. Further inclusion criteria for all participants were that they had an average intelligence score (>85 on the Hamburg Wechsler Intelligence Scale for Children or the Wechsler Adult Intelligence Scale) and that they and their parents signed the informed consent form.

Adolescents from the inpatient unit were assigned to the ED or the MDE group based on the results from the Structured Clinical Interview for DSM‐IV (SCID). Out of the 83 adolescent clinical patients who fulfilled the inclusion criteria, 75 (90%) adolescents accepted participation. All 75 participants completed the diagnostic and the attachment interview as well as the questionnaire (for a further description, see measures section) within the first 2 weeks of their inpatient treatment. Out of the 75 adolescents, 30 fulfilled the criteria for an MDE, 7 met the criteria for adjustment disorders with depressed mood (*n* = 4) or with mixed anxiety and depressed mood (*n* = 3), 30 were diagnosed with AN, 3 with BN, and 5 suffered from an ED not otherwise specified. Two of the included participants had to be excluded post hoc due to poor recording quality that did not allow a transcription of their interview, and in seven cases, the attachment interview could not be completed due to premature termination of treatment. As we wanted to focus on relatively homogenous clinical groups, we included patients with an MDE or AN and excluded patients with other ED and other depressive disorders. Our final clinical sample consisted of *n* = 30 participants with AN‐R (body mass index, *M* = 14.98, *SD* = 1.78) who had no preceding depressive episode in their medical history, and *n* = 30 participants with an MDE without any signs of ED symptomatology. In the group of patients with AN, 16 patients (53.3%) had a moderate and 14 patients (46.7%) had a severe form of AN. The average age of onset in the group of patients with AN was *M* = 13.79 (*SD* = 1.43) years, and the average duration of the illness was 10.83 months (*SD* = 6.95). Adolescents assigned to the MDE group were diagnosed with either an MDE, single episode (*n* = 22, 73.3%), or with an MDE, recurrent (*n* = 8, 26.6%). The average age of onset of the MD was 14.02 (*SD* = 1.58) years, and the average length of the episode was 13.37 months (*SD* = 13.0). Those suffering from recurrent episodes reported two (*n* = 3, 37.5%), three (*n* = 2, 25.0%), five (*n* = 2, 25.0%), and 7 (*n* = 1, 12.5%) episodes after the first onset. The majority of our adolescents from the MDE group reported symptoms on the moderate level (*n* = 20, 66.7%), and one third demonstrated a severe form (*n* = 10, 33.3%).

Although we included patients with no depressive episode in their history in our sample with AN, 16% of them took antidepressant medication. In two patients, antidepressant medicines were primarily used to reduce their purging behaviour. For the other patients, antidepressants were used to treat their severe insomnia. The general population controls (*n* = 65) were randomly selected adolescents from different parts of Austria. To exclude adolescents with a history of psychiatric or psychosomatic problems from the control group, we asked all of them about their lifetime use of psychotherapy as an inpatient or outpatient prior to the assessment procedure. We additionally conducted the screening questions of the SCID interview (Wittchen, Zaudig, & Fydrich, 1997) to gain information about their mental health status. Five adolescents were excluded from the study due to a history of psychiatric disorders in their past. The final sample consisted of 60 adolescents who did not show any signs of psychopathology on the SCID screening questions and reported that they had never been in psychiatric or psychosomatic treatment. Informed consent from all participants and their parents were obtained in accordance with the ethics committee of the Medical University of Innsbruck (AN2015‐0036).

### 2.2 Measures

#### 2.2.1 Diagnostic assessments

SCID is a semi‐structured interview to assess the following DSM‐IV Axis I disorders (SCID‐I, German translation; see Wittchen et al., 1997): affective disorders, psychotic disorders, anxiety disorders, EDs, adjustment disorders, somatoform disorders, and substance‐related disorders. The interview procedure usually takes between 1 and 2 hr depending on the level of psychopathology of the interviewee. Although the interview is usually used in adults, it was administered successfully in adolescents as well (Wittchen et al., 1997). For our study, the complete SCID interview was conducted by trained clinical psychologists at the clinic. The reliability of the SCID‐I interview for all the DSM diagnoses, and in particular for ED and major depressive disorders, can be considered as good with Kappa values above 0.70 (Zanarini et al., 2000). Furthermore, validity data are satisfactory, and the SCID‐I is often referred as the “gold standard” in determining the accuracy of mental disorders (Shear et al., 2000).

#### 2.2.2 Traumatic childhood experiences

The Childhood Trauma Questionnaire (CTQ) is an internationally accepted self‐report questionnaire to assess traumatic childhood experiences (Bernstein & Fink, 1998). The scales measure childhood maltreatment on a 5‐point Likert scale ranging from *never true* to *very often true*. Furthermore, it provides information about minimization or denial of childhood maltreatment experiences. These items are dichotomized (0 = *never* to 1 = *all other responses*). The psychometric properties have been tested in a representative German sample (*n* = 2,000). The questionnaire demonstrated good construct validity and is reliable and valid to assess childhood maltreatment retrospectively (Klinitzke, Romppel, Häuser, Brähler, & Glaesmer, 2012). According to the authors, the CTQ does not provide any norms. However, the authors have set cut scores for each type of trauma at four levels of maltreatment based on the data from nonclinical and clinical samples of adolescents and adults: none to minimal (emotional abuse: 5–8, physical abuse: 5–7, sexual abuse: 5, emotional neglect: 5–9, physical neglect: 5–7), low to moderate (emotional abuse: 9–12, physical abuse: 8–9, sexual abuse: 6–7, emotional neglect: 10–14, physical neglect: 8–9), moderate to severe (emotional abuse: 13–15, physical abuse: 10–12, sexual abuse: 8–12, emotional neglect: 15–17, physical neglect: 10–12), and severe to extreme (emotional abuse: ≥16, physical abuse: ≥13, sexual abuse: ≥13, emotional neglect: ≥18, physical neglect: ≥13).

#### 2.2.3 Attachment assessment

The AAP is a reliable and valid measure to assess adolescent and adult attachment status using an attachment theory‐based set of eight‐picture stimuli. The first picture represents a neutral scene (warm‐up stimulus) in regard to attachment, whereas the other seven pictures portray scenes of separation, solitude, death, and fear, which are considered as prominent attachment activators (Bowlby, 1969). Coders can classify the protocols into four attachment groups: secure (F), insecure‐dismissing (Ds), insecure‐preoccupied (E), and unresolved (U). Psychometric properties were tested in several studies for adolescents and adults (Buchheim et al., 2014; Gander, George, Pokorny, & Buchheim, 2016; George & West, 2012), and the AAP demonstrates a high convergent agreement with the Adult Attachment Interview but requires half of the time to administer and rate the interviews (George & West, 2011). A recent study on adult patients with chronic depression compared with healthy controls demonstrated a high convergent validity between the AAP and the AAI (*n* = 30, *κ* = 0.885, *ASE* = 0.112), *p* < 0.001, simple agreement 94% (Buchheim et al., 2018).

We analysed the following attachment‐related defences in the narratives according to the AAP manual that were developed by George and West (2011): (a) deactivation markers that move attention away from attachment distress (i.e., achievement, power, social and roles); (b) cognitive disconnection markers that refer to story actions attempting to separate emotions from attachment events (i.e., insecurities, anger, being entangled in relationships, and craving for intimacy); and (c) segregated system markers that indicate attachment‐related fear or danger that must be blocked from the consciousness as they represent a threat for the integrity of the self. Deactivation and cognitive disconnection markers are coded for each of the seven attachment‐related picture stimuli (0 = absent, 1 = present, scores ranging from 0 to 7), whereas each segregated system marker that occurs in the storylines will be added up to a sum score (≥0). Second, we counted the amount of unresolved‐specific themes in the narratives (≥0) by classifying the segregated systems into the categories found in the manual (George & West, 2012): (1) *danger/failed protection* refers to characters that are portrayed as vulnerable and endangered due to assault, abandonment, or threat; (2) *helplessness/being out of control* refers to characters that are described as despaired, chaotic, overwhelmed, or experiencing an emotional breakdown; (3) *emptiness/isolation* refers to characters that show extreme forms of aloneness by mentioning imprisonment, emptiness, worthlessness, or estrangement; (4) *spectral* is conceptualized as a dissolution of boundaries between life and death and reflected in a strong desire to disappear from this world, become invisible, or an immersion into mourning; (5) *disturbing content* refers to statements that are impossible to understand or be related to the picture as they appear extremely spooky, creepy, or they represent very odd explanations of events; (6) *obtrusions* that are evidenced by an invasion of unlicensed thoughts related to fear or threat; and (7) *constriction* refers to a blockade during story telling as the individual becomes overwhelmed by traumatic materials pulled by the picture stimuli. Furthermore, it is possible to measure differences in response to alone stimuli representing loneliness and dyadic stimuli depicting interactions in attachment relationships (George & West, 2012). These new construct‐based coding dimensions of the AAP have demonstrated highly clinical relevance and fruitful findings in adult samples to characterize traumatic dysregulation in different patient populations and settings on a deeper level (Buchheim et al., 2008; George & West, 2011). The interviews were administered by trained clinicians and psychology students, who received appropriate training. The administration training of the clinicians and students was done by providing a 4‐ to 6‐hr lecture on the AAP administration according to the AAP manual guidelines (George & West, 2011). In the next step, they had to administer six practice cases that were supervised by an experienced and certified AAP judge (M. G.). In line with the standard of all narrative‐based attachment measurements, certified judges are regarded as reliable when they have reached 80% concurrence on a minimum of 30 cases after having completed an 8‐day intensive AAP training workshop (for further information, see George & West, 2011). M. G. has used the AAP in clinical and nonclinical contexts on a weekly basis since 2013. She was also the co‐trainer for German language AAP training courses.

Although we aimed at blind administration of the AAP interviews in all patients, the AAP should be used with caution in severely traumatized adolescents (George & Buchheim, 2014). Therefore, clinicians were informed about some severe cases (*n* = 7) prior to the administration of the interview. The interviews were audiotaped and then transcribed by students and trainees at the clinic. All transcripts were afterwards rated by two certified judges (A. B. and M. G.). The second judge (A. B.) is a member of the International AAP Training Consortium and has been the principal investigator for several studies using the AAP interview in neurophysiological and clinical research since 2002. She was coder blind related to the diagnostic subgroup and the control group. Agreement for the four‐group classification (F, Ds, E, and U) was 96%, κ = 0.943 with a narrow 95% confidence interval [0.894, 0.992], *p* < 0.001, and for the two‐group classification (Secure [F] and Insecure [Ds, E, U]), it was 97%, κ = 0.907 with a narrow 95% confidence interval [0.816, 0.997], *p* < 0.001, demonstrating a good inter‐rater reliability between the two judges. Both independent coders agreed in 115 out of 120 cases. The disagreements between the coders in five cases were resolved by conference.

### 2.3 Procedure

All AAP interviews were administered in the examination rooms at the clinic (patient sample) or at the Institute of Psychology (control sample). According to the guidelines, the AAP was performed before the diagnostic interview and the questionnaire so that questions about loss, relationships, or abuse could not be assessed beforehand. Participants in the control and in the clinical groups were deemed to have intelligence scores in the mean (>85), so the Hamburg Wechsler Intelligence Scale for Children (Petermann, 2008) for adolescents under 16.11 years and the Wechsler Adult Intelligence Scale (Aster, Neubauer, & Horn, 2006) for those over 16.11 years were used to assess the IQ.

### 2.4 Analysis

First, we used Fisher's exact tests for examining differences on categorical variables like attachment classification patterns for the clinical and the control groups. Next, we conducted a one‐way analysis of variance (ANOVA) succeeded by Bonferroni post hoc tests to compare the effect of the three different groups (group of patients with an MDE, group of patients with AN, and control group) on the attachment‐related defences and unresolved themes in the AAP narratives as well as the CTQ subscales. A level of significance of a two‐sided value of *p* < 0.05 was applied to all our hypothesis tests. To calculate effect sizes, we used Cohen's (1988) conventions: *d* = 0.2 is considered as a small effect, *d* = 0.5 is a medium effect, and *d* = 0.8 is a large effect. We analysed the data using IBM SPSS statistical software for Windows (version 21.0).

## 3 RESULTS

### 3.1 Descriptive data and preliminary analyses

Sociodemographic characteristics and information on psychiatric medication of our sample (age range from 13.6 to 17.9 years) are presented in Table 1. Age distributions showed some differences across the three groups. According to an ANOVA succeeded by Bonferroni post hoc tests, our adolescents from the control group were older (*M* = 16.10, *SD* = 1.20) than adolescents with AN (*M* = 14.84, *SD* = 1.20) and with an MDE (*M* = 15.14, *SD* = 1.50), *F*(2,109) = 10.69, *p* = 0.000, η_p_^2^ = 0.156, *d* = 0.86. Even though the majority of the adolescents from the clinical and the control group were female, gender distributions reached significant differences between the three groups (clinical group girls/boys, 55/5; control group 44/16), *χ* ^2^ (2, *n* = 120) = 7.100, *p* = 0.026, Φ = 0.24. However, the two clinical groups did not differ in terms of gender (AN girls/boys, 28/2; MDE 27/3), *χ* ^2^ (1, *n* = 60) = 0.218, *p* = 0.640, Φ = 0.06. Furthermore, there were no significant differences on the living situation, *χ* ^2^ (2, *n* = 120) = 2.069, *p* = 0.355, Φ = 0.13, and the amount of siblings, *χ* ^2^ (6, *n* = 120) = 7.088, *p* = 0.313, Φ = 0.24, among the three groups. In the patient groups, less adolescents attended school, *χ* ^2^ (4, *n* = 120) = 17.039, *p* = 0.002, Φ = 0.38, and statistically more parents were single or divorced, *χ* ^2^ (4, *n* = 120) = 10.614, *p* = 0.031, Φ = 0.30. Concerning intelligence scores, the three groups showed significant differences in their mean scores, *F*(2,107) = 13.41, *p* = 0.000, η_p_^2^ = 0.200, *d* = 1.00. Post hoc analysis using Bonferroni indicated that adolescents with AN (*M* = 115.17, *SD* = 11.80) and adolescents from the control group (*M* = 116.38, *SD* = 9.17) did not differ in terms of their intelligence scores, *p* = 1.000; however, the group of patients with an MDE demonstrated significantly lower scores than the other two groups (*M* = 103.67, *SD* = 14.0), *p* = 0.001.

**Table 1:** Sociodemographic characteristics among the three groups

	AN (%)		MDE (%)		CG (%)		Φ	*p*	
	*n* = 30		*n* = 30		*n* = 60				
Gender									
Male	6.7		10.0		26.7		0.24	0.026	
Female	93.3		90.0		73.3				
Living situation									
Living alone/foster care	0.0		6.7		3.3		0.13	0.355	
Living with parents	100.0		93.3		96.7				
Amount of siblings									
Single child	23.3		13.3		6.7		0.24	0.313	
One sibling	40.0		36.7		41.7				
Two siblings	13.3		30.0		30.0				
More than two siblings	23.3		20.0		21.7				
Marital status of parents									
Married/partnership	50.0		40.0		73.3		0.30	0.031	
Single/divorced	46.7		56.7		25.0				
Deceased	3.3		3.3		1.7				
Occupation									
Attending school	76.7		70.0		96.7		0.38	0.002	
Employed/trainee	3.3		13.3		3.3				
Unemployed	20.0		16.7		0.0				
Psychiatric medication	23.3		36.7		0.0		0.44	0.000	
Antidepressants	16.7		33.3		0.0				
	*M*	*SD*	*M*	*SD*	*M*	*SD*	*F*	*df*	*p*
Age	14.84	1.20	15.14	1.50	16.10	1.20	10.69	2	0.000
Intelligence scores	115.17	11.80	103.67	14.0	116.38	9.17	13.41	2	0.000

*Note*. AN: group of patients with anorexia nervosa; MDE: group of patients with a major depressive episode group; CG: control group.

### 3.2 Attachment classifications

Results show that distribution of attachment classifications differed significantly across the three study groups (Table 2). In pairwise comparisons, the two patient groups differed from the controls, *χ* ^2^ (6, *n* = 120) = 41.887, *p* = 0.000, Φ = 0.59. In the control group, almost half of the cases were classified as F and one third of the adolescents were classified as Ds. E and U were uncommon in the control sample. In contrast to the control group, the U attachment status was overrepresented in patients with MDE and AN. Our first hypothesis could be confirmed. Although more individuals in the group of patients with AN received a U classification in the AAP compared with adolescents with MDE, this difference did not reach statistical significance, *χ* ^2^ (3, *n* = 60) = 1.909, *p* = 0.591, Φ = 0.18. Therefore, our second hypothesis was rejected.

**Table 2:** Distribution of attachment classifications for the three groups

Groups	Attachment classifications							
	F		Ds		E		U	
	*n*	%	*n*	%	*n*	%	*n*	%
AN (*n* = 30)	1	3.3	9	30.0	4	13.3	16	53.3
MDE (*n* = 30)	0	0.0	8	26.7	7	23.3	15	50.0
CG (*n* = 60)	26	43.3	20	33.3	8	13.3	6	10.0

*Note*. F: secure; Ds: insecure‐dismissing; E: insecure‐preoccupied; U: unresolved; AN: group of patients with anorexia nervosa; MDE: group of patients with a major depressive episode; CG: control group. *p* ≤ 0.05; *p* ≤ 0.001.

### 3.3 Attachment‐related defences and unresolved themes among the different groups

Significant differences were found on all three defences between the clinical and the control groups. The adolescent control group more often remained organized when confronted with stressful attachment situations by demonstrating significantly more markers of deactivation (*M* = 4.08, *SD* = 1.80) than the group of patients with AN (*M* = 3.43, *SD* = 1.60) and the group of patients with an MDE (*M* = 3.17, *SD* = 1.60), *F*(2, 117) = 3.37, *p* = 0.038, η_p_^2^ = 0.054, *d* = 0.48, and more cognitive disconnection (*M* = 6.30, *SD* = 1.78) than the two clinical groups (group of patients with AN, *M* = 5.70, *SD* = 1.42; group of patients with an MDE, *M* = 5.50, *SD* = 1.48), *F*(2, 117) = 5.19, *p* = 0.007, η_p_^2^ = 0.082, *d* = 0.60). Yet interestingly, the amount of segregated systems did not reach statistical significance between the control (*M* = 8.62, *SD* = 5.73) and the clinical groups in general (group of patients with AN, *M* = 8.73, *SD* = 6.29; group of patients with MDE, *M* = 6.80, *SD* = 5.05), *F*(2, 117) = 1.20, *p* = 0.306, η_p_^2^ = 0.020, *d* = 0.29, suggesting that attachment‐related threats can be found in the narratives of patients and controls to the same extent. Therefore, we needed to delve into the unresolved category in more detail to extend our understanding of disorder‐specific nuances related to attachment trauma. Compared with the control group who rather managed to deal with attachment‐related distress, we found that the segregated systems were more often associated to attachment dysregulation in the clinical group, *F*(2, 117) = 9.27, *p* = 0.000, η_p_^2^ = 0.137, *d* = 0.80.

Furthermore, our findings reveal significant differences between the three groups on the amount of unresolved specific themes in the attachment narratives. A one‐way between‐subjects ANOVA demonstrates that the clinical groups showed more dysregulation when confronted with themes of danger/failed protection, emptiness/isolation, and spectral elements (Table 3). Post hoc comparisons using Bonferroni correction indicate that anorexic patients (*M* = 1.47, *SD* = 1.36) also showed more markers of isolation and emptiness in their attachment narratives than adolescents with MDE (*M* = 0.70, *SD* = 1.49), *F*(2,117) = 3.11, *p* = 0.034, η_p_^2^ = 0.050, *d* = 0.46. These markers were more often associated to attachment dysregulation, especially when characters were confronted with extreme loneliness in alone picture stimuli (group of patients with AN, *M* = 0.47, *SD* = 0.78; group of patients with an MDE, *M* = 0.13, *SD* = 0.57), *F*(2,117) = 8.54, *p* = 0.028, η_p_^2^ = 0.127, *d* = 0.76. Assuming that adolescents with AN show more dysregulation in the context of isolation, it is no surprise that their wish to disappear from this world or to become invisible might be their solution to deal with their solitude (Latzer & Hochdorf, 2005). In our study, this is evidenced by a higher amount of unresolved spectral markers in the attachment narratives of patients with AN (*M* = 0.33, *SD* = 0.96) compared with those of the adolescents with MDE (*M* = 0.07, *SD* = 0.25), *F*(2,117) = 4.11, *p* = 0.042, η_p_^2^ = 0.066, *d* = 0.65.

**Table 3:** Mean scores on the occurrence of unresolved‐specific attachment themes in response to all picture stimuli among the clinical and the control groups

	AN		MDE		CG				
	*n* = 30		*n* = 30		*n* = 60				
	*M*	*SD*	*M*	*SD*	*M*	*SD*	*F*	*df*	*p*
Danger/failed protection	1.33	2.75	1.07	1.65	0.13	0.50	6.58	2	0.002
Helplessness/being out of control	0.37	1.03	0.60	1.00	0.20	0.94	1.69	2	0.189
Emptiness/isolation	0.47	0.78	0.13	0.57	0.17	0.02	8.54	2	0.000
Spectral	0.33	0.96	0.07	0.25	0.02	0.13	4.11	2	0.019
Constriction	0.00	0.00	0.07	0.37	0.00	0.00	1.51	2	0.225

*Note*. AN: group of patients with anorexia nervosa; MDE: group of patients with a major depressive episode; CG: control group. *p* ≤ 0.05; *p* ≤ 0.001.

### 3.4 Attachment and childhood trauma

Our results demonstrate that both clinical groups reported mean scores on the subscales emotional abuse, emotional neglect, and physical abuse on the moderate to severe range, whereas on the subscales physical neglect and sexual abuse, they reported mean scores in the low to moderate range. The control group, on the other hand, scored low on all CTQ subscales. ANOVA revealed a main group effect on the CTQ subscale emotional abuse, *F*(2, 117) = 8.457, *p* = 0.000, η_p_^2^ = 0.126, *d* = 0.77, emotional neglect, *F*(2, 117) = 8.553, *p* = 0.000, η_p_^2^ = 0.128, *d* = 0.76, and physical abuse, *F*(2,117) = 3.134, *p* = 0.047, η_p_^2^ = 0.051, *d* = 0.46 (Table 4). Post hoc analyses using Bonferroni correction furthermore show that adolescents with MDE reported higher scores on the two subscales emotional abuse (group of patients with MDE, *M* = 11.73, *SD* = 5.63; group of patients with AN, *M* = 9.17, *SD* = 4.79, *p* = 0.028) and emotional neglect (*M* = 12.43, *SD* = 4.64; group of patients with AN, *M* = 9.23, *SD* = 4.31 *p* = 0.005) than patients with AN. To test whether participants underreport maltreatment, the CTQ measure includes a minimization/denial scale that consists of three items: (1) *There was nothing I wanted to change about my family*; (2) *I had the perfect childhood*; and (3) *I had the best family in the world*. Yet interestingly, our findings show that the group of patients with AN scored higher on the minimization items than the group of patients with an MDE. Although these differences did not reach statistical significance for most of the minimization items, patients with AN reported significantly more often that they had the best family in the world, *F*(2, 117) = 3.682, *p* = 0.028, η_p_^2^ = 0.059, *d* = 0.5. Post hoc analysis indicated that patients with AN scored higher on this item (*M* = 0.40, *SD* = 0.50) than adolescents with MDE (*M* = 0.17, *SD* = 0.38, *p* = 0.049).

**Table 4:** Mean scores on the CTQ subscales and items for minimization/denial for the clinical and the control groups

	AN		MDE		CG				
	*n* = 30		*n* = 30		*n* = 30				
	*M*	*SD*	*M*	*SD*	*M*	*SD*	*F*	*df*	*p*
Emotional abuse	9.17	4.79	11.73	5.63	7.63	3.56	8.46	2	0.000
Physical abuse	5.90	3.34	6.63	3.66	5.23	0.79	3.13	2	0.047
Sexual abuse	5.97	3.69	5.53	1.63	5.27	0.97	1.09	2	0.340
Emotional neglect	9.23	4.31	12.43	4.64	8.48	4.16	8.55	2	0.000
Physical neglect	6.67	3.12	7.00	2.45	6.30	2.14	0.82	2	0.443
Minimization/denial (total)	1.07	1.36	0.57	1.04	1.08	1.23	1.98	2	0.143
Minimization: There was nothing I wanted to change about my family	0.37	0.49	0.20	0.41	0.28	0.45	1.02	2	0.365
Minimization: I had the perfect childhood	0.30	0.47	0.20	0.41	0.35	0.48	1.06	2	0.35
Minimization: I had the best family in the world	0.40	0.50	0.17	0.38	0.45	0.50	3.68	2	0.028

*Note*. AN: group of patients with anorexia nervosa; MDE: group of patients with a major depressive episode; CG: control group; CTQ: Childhood Trauma Questionnaire. Minimization items are dichotomized ranging from 0 *= never* to 1 = *all other responses*.

## 4 DISCUSSION

The aim of this study was to investigate attachment in adolescents with AN and MDE in terms of classification distribution, disorder‐specific attachment themes, and subjectively reported childhood maltreatment.

In line with results from adult studies (Bakermans‐Kranenburg & van IJzendoorn, 2009; Juen et al., 2013), we found an overrepresentation of the unresolved pattern in our clinical adolescent sample. We classified 53% of our adolescents with AN and 50% of adolescents with MDE as unresolved. Concerning AN, Delvecchio et al. (2014) found that almost half of the adult patients with AN were classified as unresolved and 37% were classified as insecure‐dismissing in the AAP. Ward et al. (2001) reported similar results of 50% unresolved cases in their sample of adult patients with AN. In the Ringer and Crittenden (2007) study, the rate was a bit lower where one third of their ED (AN, BN, and EDNOS) sample received an unresolved attachment pattern. One reason for this lower rate as reported in the Ringer and Crittenden (2007) study could be that their sample consisted of inpatient and outpatients with different subtypes of ED, whereas the previously mentioned studies focus on groups of inpatients with AN. Concerning MDE, our prevalence rate of the unresolved attachment pattern in adolescents is consistent with research findings on adult chronic depression and children with depressive symptoms in the clinical‐range, where more than half of the patients were classified as unresolved (Fonagy et al., 1996; Juen et al., 2013). However, our rate differs from other research findings that report lower rates in adults with depression (West & George, 2002). These authors suggest that child rearing or interpersonal issues might be more relevant for adult patients than abuse or attachment trauma (Bakermans‐Kranenburg & van IJzendoorn, 2009; West & George, 2002). Yet more research is needed before conclusions can be drawn regarding specific links between attachment patterns and the different subtypes of depressive disorders in adolescence and adults.

The high prevalence rate of the unresolved attachment pattern in our adolescent clinical population in general might be due to several reasons. First, our sample consisted of inpatient adolescents with moderate to severe psychopathology, whereas some adult studies conducted their research in mixed inpatient and outpatient samples (De Paoli et al., 2017; Ringer & Crittenden, 2007) including participants that have currently recovered from their disorder (De Paoli et al., 2017). Second, some researchers propose that adolescents are probably exposed to more adverse life events and stressors related to interpersonal and structural contexts (i.e., changes in school settings, first experiences of romantic relationship breakups, family conflicts, or the death of a grandparent) than individuals at other time points of the human life span (Aikins, Howes, & Hamilton, 2009). These events might affect adolescents to a greater extend as they probably have not yet developed elaborated coping strategies and skills to manage these difficulties. At the same time, their striving for autonomy makes it more difficult for them to seek their caregiver's shoulder to cry on in situations of extreme attachment‐related stress and thus probably leaves them in a state of dysregulation (Aikins et al., 2009). It could also be argued that in contrast to other studies with a minority of patients demonstrating past experiences of trauma (West & George, 2002), our inpatient adolescent sample reported traumatic experiences in the moderate to severe range, which might be associated with a significant unresolved attachment bias.

By looking at disorder‐specific themes that were associated to the unresolved category, this study is building on the growing body of literature examining attachment issues in clinical work with adolescents (Kobak & Kerig, 2015). We observed that the clinical and control group did not differ on the amount of segregated systems in the attachment narratives per se. This is in line with the findings from other researchers (Buchheim et al., 2008; Buchheim & George, 2001), who argued for an in‐depth analysis of traumatic contents and defensive mechanisms to extend our understanding of the unresolved pattern in psychiatric patients and transfer its relevance into the psychotherapeutic practice (George & Buchheim, 2014). In this respect, our control group demonstrated more thoughtful self‐exploration and confidence in their attachment figures availability to remain organized, whereas the clinical group more often demonstrated attachment dysregulation. Furthermore, the control group employed more adaptive defences to divert or split attention away from event‐related feelings and memories in order to keep the attachment system organized (Solomon & George, 2011). In contrast to other studies in adults that found more use of deactivation in patients with major depressive disorders (Dozier et al., 2008; Juen et al., 2013), adolescents with MDE and AN demonstrated an equally low amount of defensive strategies in their AAP narratives. These results suggest that younger age groups might have more difficulties to remain organized than adult patients (Dozier et al., 2008).

One of the most striking results of the present study was that the group of patients with AN demonstrated many unresolved themes that indicate dissolution of boundaries between life and death in response to the AAP picture stimuli. Research addressing the question of life and death in patients with ED (Latzer & Hochdorf, 2005) suggests that patients with AN are considered being not so much attracted to death itself but rather showing a rejection of life. As they seem to be unable to gain control over their lives, especially in situations of extreme loneliness and isolation, their symptoms might represent a kind of latent suicidal act in which they gain a false feeling of control over their situation (Latzer & Hochdorf, 2005). The attachment narratives of our group of adolescents with AN revealed a similar picture. In their stories, individuals often felt desperately alone, empty, and separated from the world especially when attachment relationships were threatened, distressed, or broken. In these situations, feelings of isolation became too overwhelming, and their wish to disappear from this world or to be invisible became apparent. Research found that patients with ED often lack confidence to cope with negative emotions (Burns et al., 2012; Svaldi, Griepenstroh, Tuschen‐Caffier, & Ehring, 2012) and cannot rely on attachment figures for comfort and safety (Orzolek‐Kronner, 2002; Ringer & Crittenden, 2007). They often do not dare to express their feelings, interests, and needs in an appropriate manner (Burns et al., 2012) but rather try to exist within the narrowest parameter (Strober, 1991). In this sense, their control over their eating behaviour and their resentment of food might provide them with a false sense of control over their lives and could serve as a replacement for their secure base needs (Latzer & Hochdorf, 2005).

Our results on subjectively reported childhood experiences might also provide some support for this hypothesis. We found moderate to high levels on emotional abuse and emotional neglect as well as physical abuse on the CTQ subscales in those with AN and those with MDE. Studies showed that patients with ED often report interpersonal trauma and adverse childhood circumstances including emotional abuse, parental mental health problems, or divorce (Lejonclou et al., 2014). In patients with BN, AN binge‐eating/purging type, or BED, researchers also found higher rates of sexual trauma, but in line with our findings, this is not reported in patients with AN restrictive type (Brewerton, 2007; Jaite et al., 2012). Yet our findings reveal that, compared with adolescents with MDE, patients with AN reached higher scores on the minimization/denial scale, especially on questions regarding family issues. This is particularly interesting as minimization of the illness and interpersonal difficulties are often discussed as primary features of patients with ED (Couturier & Lock, 2006). Given the high prevalence of unresolved cases in our adolescent sample, their denial of ruptures in the parent–child attachment bond might serve as a form of self‐protection at the cost of an open communication of their inner feelings. In his context, their eating behaviour might be a maladaptive way to regain control and prevent abandonment in situations where attachment relationships are threatened, distressed, or broken (Latzer & Hochdorf, 2005). This might also explain why adolescents with AN often idealize their parents and describe their mothers as great facilitators of independence (Fonagy et al., 1996; Orzolek‐Kronner, 2002). However, it seems that in adulthood, this view on parental relationships changes to a more problematic mother–daughter relationship that is characterized by a role‐reversal behaviour resulting in a greater display of anger, confusion, and demand for the caregiver's compliance in daughters with ED (Barone & Guiducci, 2009; Ringer & Crittenden, 2007). In this respect, some authors discuss whether it is not solely the traumatic experiences in daughters but probably also in mothers that are crucial for the ruptures in the attachment relationship (Ringer & Crittenden, 2007; Ward et al., 2001). That is, mothers probably have their own history of traumatic experiences in their past (Shoebridge & Gowers, 2000), and even though they seek a special closeness with their children, they might retreat and become unavailable when their children need them the most. To date, we know hardly anything about this transgenerational transmission of unresolved attachment pattern in clinical groups, and thus, these issues represent an exciting research area especially in the field of adolescent psychopathology.

Additionally, it seems that the role of fathers as primary caregivers has been neglected in narrative‐based attachment studies. In the field of AN, findings from researchers using self‐report measurements of attachment report that patients with AN describe their fathers as highly critical, less caring, and more controlling (Orzolek‐Kronner, 2002). Furthermore, results indicate a paternal tendency to be emotionally absent, cold, and defensive in response to their daughter's illness (Ballotin, Mannarini, Mensi, Chiappedi, & Gatta, 2017). As a result, patients with AN might feel more alienated from their fathers (Gander et al., 2015; Horesh, Sommerfeld, Wolf, Zubery, & Zalsman, 2015; Orzolek‐Kronner, 2002), which consequently affects the quality of family interactions and the treatment outcome (Ballotin et al., 2017). A closer investigation of traumatic experiences and unresolved attachment status in fathers might help to gain a better understanding of underlying mechanisms that cause these defensive responses in fathers. For clinical practice, these results might help to accomplish a better paterinal involvement characterized by warmth and participation, which is considered to be crucial for an adolescent's positive treatment outcome (Ballotin et al., 2017).

Adolescents with MDE also displayed a fair amount of isolation, danger, and failed protection in their attachment narratives. Furthermore, they reported the highest levels of emotional neglect and abuse in their childhood; however, in contrast to patients with AN, they did not underreport or deny these experiences. Their attachment stories often portrayed characters as vulnerable and unable to find or receive protection from their attachment figures when facing severe conditions like physical assault, various forms of abuse (i.e., physical, sexual, or emotional), or life‐threatening events. In these situations, characters feel abandoned, helpless, and frustrated, which leaves them in a state of attachment dysregulation. Our results might contribute to the ongoing discussion of psychological dynamics that underlie the possible links between preoccupied attachment patterns and depression. Attachment researchers have increasingly found that patients with depression often experienced contradictory or unpredictable caregiver responses (West & George, 2002) that might result in a child's failure to coherently integrate attachment‐related experiences, affect, and memories (Bowlby, 1969). From this perspective, depressive symptoms could be viewed as a basic reaction to the sense of frustration when these adolescents fail to actively explore their internal models of attachment, to use attachment relationships for comfort and safety, and to be able to take specific actions to remove from attachment‐related dysregulation (West & George, 2002).

The previously mentioned results on attachment and experiences of childhood maltreatment shed light on possible mechanisms that might be associated to the onset of psychopathology in adolescence. Although there are some recently developed models that explain how attachment mediates the relationship between traumatic childhood experiences and later psychopathology (Tasca et al., 2013; Tasca & Balfour, 2014), they are not concerned with the unresolved status but rather conceptualize attachment dimensionally on a continuum between attachment avoidance and attachment anxiety. Our results might provide a good framework to expand these models by taking a closer look at the unresolved status and attachment‐related traumatic material. Future research elaborating an expanded model that includes these aspects might contribute to a better understanding of how attachment‐related aspects could mediate early trauma and adolescent psychopathology.

The present study has a number of strengths: First, to our knowledge, it is the only study to date that has used a narrative attachment measurement in a clinical adolescent sample with AN and MDE (Gander et al., 2015). In this respect, our sample size of 120 adolescents is comparable with those found in studies using self‐report measurements of attachment. In narrative‐based research, smaller samples are more common (Bakermans‐Kranenburg & van IJzendoorn, 2009; Ravitz et al., 2010), especially in psychiatric patients (Fonagy et al., 1996; Ward et al., 2001), as the interview procedure can be time‐consuming in administration and coding. Second, we investigated the unresolved status in more detail for the first time by identifying disorder‐specific attachment themes that put individuals at risk of becoming disorganized and dysregulated. Third, as preliminary research findings suggest differences between clinical and healthy groups in adults (Delvecchio et al., 2014), we expand these results by focusing on group differences among two adolescent clinical groups and study whether or not these differences persist among patients with severe psychopathology. Fourth, although clinical case studies (Dallos & Denford, 2008; Ringer & Crittenden, 2007) reveal some important areas of unresolved and traumatic processes in the family history, no research has assessed subjectively reported traumatic childhood experiences in relation to attachment representations.

Despite the strengths of this study, it has several limitations. First, our clinical sample consisted of adolescent inpatients. Some of these patients might show more symptom severity and a higher comorbidity rate than outpatient samples. Although the sociodemographic data and the clinical symptoms of our study are comparable with the majority of AAI/AAP studies (see,e.g., Fonagy et al., 1996, Delvecchio et al., 2014, or Juen et al., 2013, who recruited their samples in psychiatric inpatient units and reported moderate to severe levels of psychopathology), other studies include patients who have currently recovered from their disorder (De Paoli et al., 2017) or who were not diagnosed with a psychiatric disorder according to DSM criteria (Burns et al., 2012). Furthermore, some studies did not only recruit their sample in hospitals but by advertisement or from private clinical psychologists (De Paoli et al., 2017; Ringer & Crittenden, 2007). Thus, future research might do well to differentiate inpatient and outpatient adolescent samples for symptom severity and for possible comorbidity rates as these variables might have a significant impact on attachment‐specific characteristics. In this respect, it would also be interesting to discriminate between subtypes of ED and major depressive disorders in order to draw further conclusions on disorder‐specific attachment characteristics. Furthermore, we used the consecutive sampling method for our study. We aimed to include all accessible inpatient adolescents that met the inclusion criteria (regardless of their symptom severity, etc.) to reach a better representation of the entire inpatient population. The time period for this study was 3 years to avoid potential bias like, for example, seasonal variations in the MDEs. However, using this sampling method also implies that our sample might not represent the entire clinical population accurately. Therefore, our research results do not allow generalizations pertaining to the entire adolescent clinical population.

Second, the control sample was made up largely of middle‐class subjects with a limited ethnic diversity. Whereas sociodemographic variables like educational levels, gender, or age do not seem to be associated with attachment classifications in adolescents (Gander et al., 2016), our results may not be generalizable to populations with different ethnic backgrounds. Furthermore, most of the parents from the clinical group were divorced, whereas the majority of parents from the control group were married. Although the low percentage of married parents is quite common in clinical populations, future research might do well to replicate these findings in adolescents with similar family circumstances. A final limitation was that our AAP coding was not completely blinded. Researchers have stated before that it is almost impossible to achieve complete blindness towards the diagnosis in clinical attachment research using narrative techniques like the Adult Attachment Interview (Barone & Guiducci, 2009) because the interview procedure reveals information on the patients' life histories. In addition, the majority of studies in this field does not state whether coders were blind to the patients' diagnoses but rather focus on the fact that the coders rated the transcripts independently (Delvecchio et al., 2014; Ward et al., 2001). The AAP is not an autobiographical interview and thus circumvents this limitation in attachment research. In line with other researchers, all transcripts were rated independently by both coders, and we maintained the blindness of one coder; however, the second coder was informed of some severe cases and thus was not blind to all cases.

The clinical implications of our findings on attachment‐related characteristics and childhood trauma in adolescents with AN and MDE affect several practice areas. In terms of psychological assessment, using a narrative‐based attachment measurement provides clinicians with a new insight into the patients' problems, attachment‐related defensive structures, and unresolved mental states related to trauma or loss (Delvecchio et al., 2014; George & Buchheim, 2014; George & West, 2011). It might be clinically useful to assess these patients for a history of potentially traumatic events to gain further information about the occurrence, type, and frequency of possible traumatic experiences in the past. A qualitative analysis of traumatic material that underlies the unresolved attachment status might lay out the foundation for subsequent attachment‐based therapeutic practices that help patients to recognize the impact of traumatic experiences on their current affect regulation, interpersonal functioning, and psychopathological symptoms (George & Buchheim, 2014; O'Shaughnessy & Dallos, 2009). For example, the narratives of our patients with AN reveal that feelings of isolation take centre stage when confronted with threatened abandonment or ruptures in their relationships. Their disturbed eating behaviours might serve as a maladaptive coping strategy to gain control over threatened separation. However, this can only happen at the cost of an inappropriate communication of emotional states and an impaired autonomy development (Orzolek‐Kronner, 2002), leaving them in a state of dysregulation that is characterized by invisibility and disappearance. By fostering a secure therapeutic relationship (Burke, Danquah, & Berry, 2016), clinicians can assist their adolescent patients in revealing and facing these and other disorder‐specific unresolved themes when confronted with attachment stressors and support them to develop more adaptive emotion regulation strategies related to their unresolved attachment status. In the context of psychotherapy, attachment theory provides a good framework for clinicians to formulate attachment‐related goals for treatments based on the individual attachment characteristics (George & West, 2011).

Additionally, the use of narrative‐based attachment instruments in clinical settings might help practitioners to understand how attachment‐related dynamics are activated in their patients' daily lives and in family therapeutic settings (Slade, 2008) and to anticipate the nature of the therapeutic alliance with their patients (Lis, Mazzeschi, Di Riso, & Salcuni, 2011). Developing intervention strategies tailoring to disorder‐specific attachment characteristics and potentially traumatizing material hold good promise to improve the outcome and minimize the risk for therapy drop‐out in adolescent patients with psychiatric disorders who experienced early trauma.

Our findings illuminate in‐depth details on attachment‐related trauma in adolescents with AN and MDE for the first time. The results might represent the foundation for future studies on the transgenerational transmission of attachment trauma and its relevance for the maintenance of AN and MDE into adulthood. Clinicians who treat adolescents with a history of trauma might benefit from assessing their attachment patterns as they provide a far‐reaching insight into the psychological functioning within and beyond the family system.

## CONFLICT OF INTEREST

The authors declare that this research did not receive any specific grant from funding agencies in the public, commercial, or not‐for‐profit sectors.
